# Proteins altered by elevated levels of palmitate or glucose implicated in impaired glucose-stimulated insulin secretion

**DOI:** 10.1186/1477-5956-7-24

**Published:** 2009-07-16

**Authors:** E-ri M Sol, Meri Hovsepyan, Peter Bergsten

**Affiliations:** 1 Department of Medical Cell Biology, Uppsala University, Uppsala, Sweden; 2 Institute of Molecular Biology of Armenian National Academy of Sciences, Yerevan, Armenia

## Abstract

**Background:**

Development of type 2 diabetes mellitus (T2DM) is characterized by aberrant insulin secretory patterns, where elevated insulin levels at non-stimulatory basal conditions and reduced hormonal levels at stimulatory conditions are major components. To delineate mechanisms responsible for these alterations we cultured INS-1E cells for 48 hours at 20 mM glucose in absence or presence of 0.5 mM palmitate, when stimulatory secretion of insulin was reduced or basal secretion was elevated, respectively.

**Results:**

After culture, cells were protein profiled by SELDI-TOF-MS and 2D-PAGE. Differentially expressed proteins were discovered and identified by peptide mass fingerprinting. Complimentary protein profiles were obtained by the two approaches with SELDI-TOF-MS being more efficient in separating proteins in the low molecular range and 2D-PAGE in the high molecular range. Identified proteins included alpha glucosidase, calmodulin, gars, glucose-6-phosphate dehydrogenase, heterogenous nuclear ribonucleoprotein A3, lon peptidase, nicotineamide adenine dinucleotide hydrogen (NADH) dehydrogenase, phosphoglycerate kinase, proteasome p45, rab2, pyruvate kinase and t-complex protein. The observed glucose-induced differential protein expression pattern indicates enhanced glucose metabolism, defense against reactive oxygen species, enhanced protein translation, folding and degradation and decreased insulin granular formation and trafficking. Palmitate-induced changes could be related to altered exocytosis.

**Conclusion:**

The identified altered proteins indicate mechanism important for altered β-cell function in T2DM.

## Background

Reduced insulin secretory capacity and loss of mass of the pancreatic β-cells are hallmarks in the development of type 2 diabetes mellitus (T2DM) [1,2]. The aberrant insulin secretory characteristics have been linked to exposure of the cells to prolonged periods of elevated glucose and fatty acid levels [3]. The secretory alterations depend on what fatty acid is present during exposure and include enhanced secretion at basal glucose concentrations and/or lowered insulin levels at elevated glucose concentrations [3,4]. These alterations lead to reduced or even abolished glucose-stimulated insulin secretion (GSIS). In addition, β-cell mass is reduced by the combined elevation of glucose and fatty acids [5]. The fatty acid palmitate has especially negative effects on both β-cell function and mass via different mechanisms [4,6-10]. Despite these efforts to define causes responsible for the altered insulin secretory pattern observed after exposure to elevated levels of glucose and palmitate, underlying mechanisms remain to a large extent undefined. In an attempt to further understand how elevated levels of the two nutrients cause the secretory alterations and taking the polygenicity of obesity-induced type 2 diabetes mellitus into account [11], insulin-producing INS-1E cells were cultured in the presence of elevated levels of glucose and palmitate and protein profiled. In addition, INS-1E cells over-expressing the fatty acid transporter carnitine palmityoltransferase 1 (CPT1) were also protein profiled. The latter approach was inspired from recent results, where improvement of insulin release in INS-1E cells exposed to palmitate was achieved by redirecting the fatty acid from esterification to oxidation by over-expressing CPT1 in INS-1E cells [12].

In previous work we have employed SELDI-TOF-MS and 2D-PAGE for β-cell protein profiling [13,14]. Given the advantages but also limitations of the approaches [15], in the present study we employed both strategies to determine protein patterns of insulin-producing cells with aberrant secretory pattern as a result of exposing the cells to either elevated levels of glucose or palmitate. The study allowed methodological comparisons and identification of β-cell proteins differentially expressed by palmitate or glucose, which may represent mechanisms by which the nutrients negatively affect GSIS.

## Results

### Glucose-stimulated insulin secretion and insulin content of INS-1E cells

**Figure 1:**
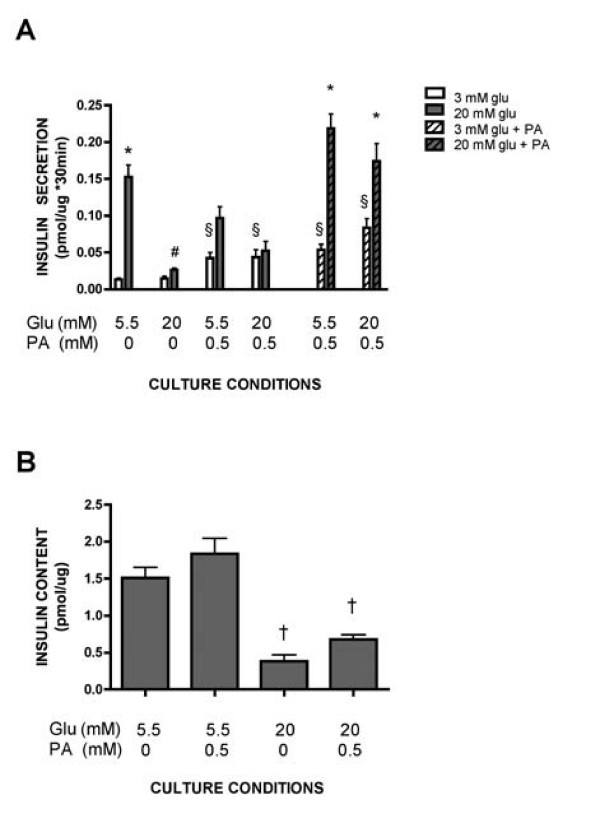
**Insulin secretion from gluco- and lipotoxic INS-1E cells**. Insulin release (A) and content (B) from INS-1E cells cultured at 5.5 or 20 mM glucose (Glu) in the absence or presence of 0.5 mM palmitate (PA) for 48 hours. Insulin secretion was measured by acutely stimulating the cells with 3 mM (white bars), 20 mM glucose (grey bars), 3 mM glucose and palmitate (white hatched bars) or 20 mM glucose and palmitate (grey hatched bars) for 30 minutes after culture at the indicated culture conditions. Insulin release and content were normalized to total protein. Results are means ± SEM for four to six independent experiments. *p < 0.05 compared to basal insulin release, #p < 0.05 compared to stimulatory insulin release from cells cultured at 5.5 mM glucose, §p < 0.05 compared to basal insulin release from cells cultured at 5.5 or 20 mM glucose, †p < 0.05 denotes effect of 20 mM glucose.

INS-1E cells were cultured for 48 hours in the presence of 5.5 or 20 mM glucose in the absence or presence of palmitate. After culture, GSIS was determined by measuring insulin release in response to 3 (basal) or 20 (stimulatory) mM glucose during 30 min (Fig 1A). In cells cultured in the presence of 5.5 mM glucose alone, insulin release during the 30-minute period increased more than 10-fold when insulin released at 3 mM glucose was compared with that released at 20 mM. When the culture glucose concentration was raised to 20 mM glucose, insulin release during the 30-minute period in the presence of the stimulatory glucose concentration (20 mM) was reduced to such an extent that it was not different from that released from cells acutely exposed to the basal glucose concentration (3 mM). When palmitate was included during culture of cells exposed to 5.5 or 20 mM glucose, insulin release during the subsequent 30-minute period in response to 3 mM glucose was enhanced. In addition, insulin released during the 30-minute period in response to 20 mM glucose was reduced. As a consequence the difference between basal and stimulatory insulin release was decreased especially in cells cultured in the presence of 20 mM glucose and palmitate.

Insulin release from cells cultured in the presence of 5.5 or 20 mM glucose and palmitate was also measured during the 30-minute period following the culture in the continued presence of the fatty acid. Under these conditions insulin secretion in response to 20 mM glucose during the 30-minute period was similar to that observed from control cells cultured in the presence of 5.5 mM glucose alone. Despite the augmented stimulatory insulin secretion, GSIS was still impaired since basal insulin release during the 30-minute period was still elevated. In view of these changes in insulin release we conclude that whereas impaired GSIS from INS-1E cultured in the presence of elevated levels of glucose alone was the result of reduced stimulatory insulin release, impaired GSIS in the presence of palmitate during the culture period resulted in enhanced basal insulin release.

Insulin content of INS-1E cells cultured for 48 hours was measured. Whereas inclusion of palmitate did not affect insulin content of INS-1E cells, increasing the culture glucose concentration from 5.5 to 20 mM reduced insulin content (Fig 1B).

### Elevation of basal insulin release: differential INS-1E protein expression induced by palmitate

**Figure 2:**
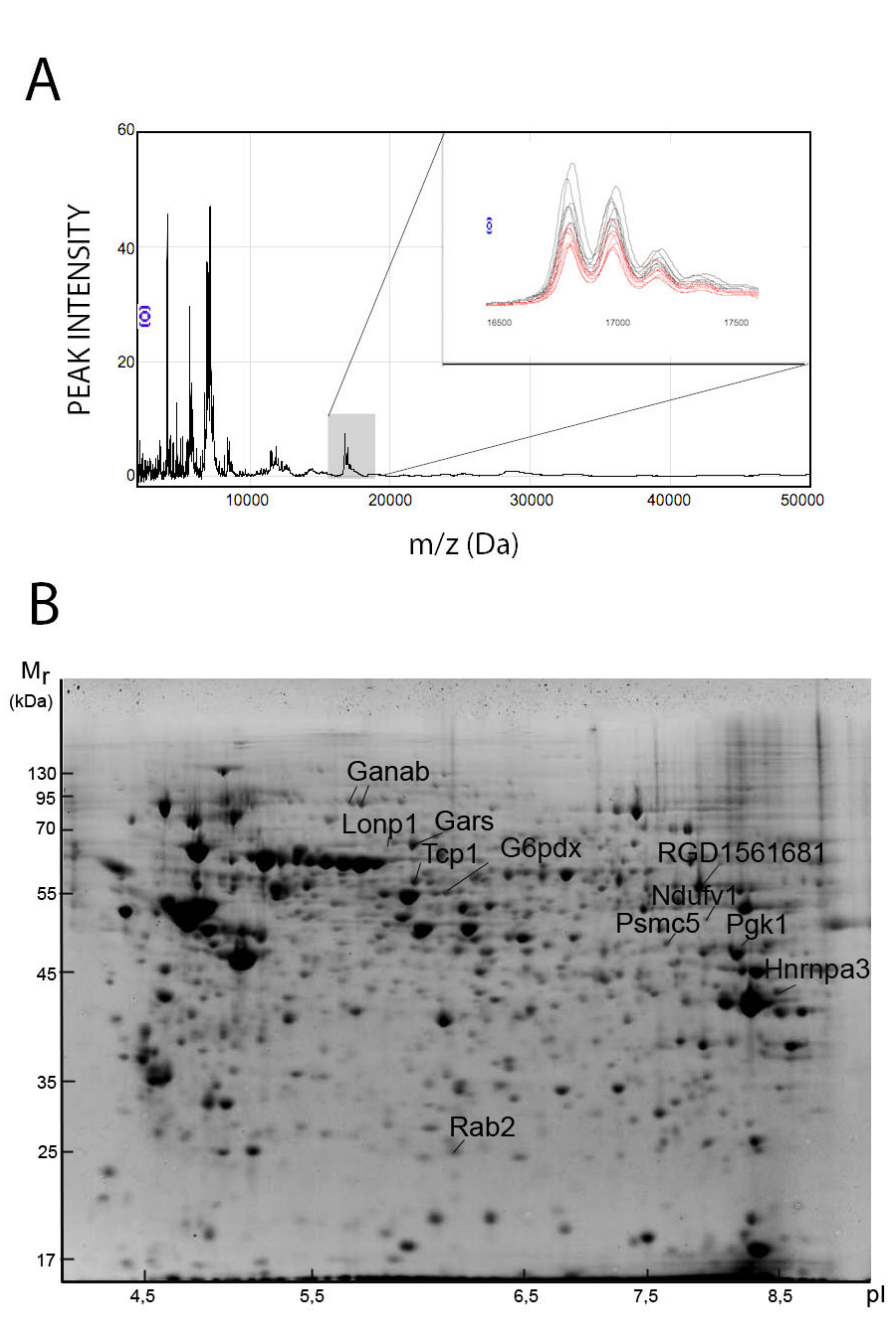
**INS-1E cell SELDI-TOF mass spectra and 2D-PAGE map**. SELDI-TOF mass spectrum (A) and 2D-PAGE map (B) generated from INS-1E cells cultured at 20 mM glucose in the absence or presence of 0.5 mM palmitate for 48 hours. Representative mass spectrum with magnified area of calmodulin with the tentative masses of 16.8, 17.0 and 17.2 kDa, (n = 6) from cells cultured at 20 mM glucose alone (black traces) or presence of palmitate (red traces). Identified proteins are marked in the gel image by their gene names. M_r_, m/z and pI denote relative molecular mass, mass over charge and isoelectric point of the protein.

To elucidate mechanisms by which prolonged exposure to palmitate increase basal insulin release, INS-1E cells were cultured in the absence or presence of palmitate at 20 mM glucose. After culture, protein profiles were generated by SELDI-TOF-MS and 2D-PAGE. Altogether 28 palmitate-regulated proteins were discovered, 11 by the former and 17 by the latter approach (Table 1). Three proteins, which were down-regulated by palmitate and had tentative masses of 16.8, 17.0 and 17.2 kDa, were located in a part of the mass spectrum with less frequent other peaks (Fig 2A). The proteins were purified and identified as calmodulin (Table 2 and 3).

**Table 1:** Palmitate-regulated INS-1E proteins

**Protein mass (kDa)**	**pI**	**Protein expression ratio (G20+PA)/G20**
5.74		1.59

5.81		1.30

7.14		1.65

7.22		1.29

7.32		1.62

7.40		1.37

11.87		0.82

16.80 ^Calm1, Calm3^		0.71

16.98 ^Calm1, Calm3^		0.72

17.18 ^Calm1, Calm3^		0.72

78.28		1.44

12	5.5	0.57

35	6.5	1.17

36	5.4	0.76

37	9.5	0.88

38	6.1	0.85

38	9.5	1.45

41	7.7	0.97

41	7.9	1.15

41	9.0	0.73

44	5.1	1.85

46	5.0	1.37

49	5.0	1.75

50	8.5	1.54

53	5.9	3.3

61	6.8	0.86

81	6.9	2.09

118	4.7	1.34

INS-1E cells were cultured at 20 mM glucose in the absence or presence of palmitate for 48 hours. After culture, cells were lysed and protein profiles obtained by SELDI-TOF-MS using Q10-array or by 2D-PAGE. Masses of differentially expressed proteins by SELDI-TOF-MS (11 first proteins) or by 2D-PAGE are listed. Gene names of identified proteins are indicated. Results are means ± SEM 4–6 independent experiments.

**Table 2:** Glucose and palmitate-regulated INS-1E proteins

**Gene name**	**Protein name**	**Synonyms**	**M_r _(Da)**	**pI**
Calm1, Calm3	Calmodulin		16696	4,09
G6pdx, G6pd	Glucose-6-phosphate 1-dehydrogenase	G6PD EC 1.1.1.49	59794	5,97
Ganab	Alpha glucosidase 2 alpha neutral subunit		90857	5,77
Gars	Gars protein		72669	5,76
Hnrnpa3, Hnrpa3	Heterogeneous nuclear ribonucleoprotein A3	hnRNP A3	37291	8,46
Lonp1, Lon, Prss15	Lon peptidase 1	EC 3.4.21.-, Lon protease-like protein LONP, Mitochondrial ATP-dependent protease Lon, Serine protease 15	106296	6,17
Ndufv1	NADH dehydrogenase (ubiquinone) flavoprotein 1		51383	8,37
Pgk1, Pgk-1	Phosphoglycerate kinase 1	EC 2.7.2.3	44909	8,02
Psmc5	Proteasome p45/SUG		42174	7,11
Rab2	RAB2, member RAS oncogene family		18513	6,43
RGD1561681_predict.	Pyruvate kinase 3		58312	7,15
Tcp1, Cct1, Ccta	t-complex protein 1	CCT-alpha, TCP-1-alpha	60835	5,86

INS-1E cells were cultured at 5.5 or 20 mM glucose in the absence or presence of palmitate for 48 hours. After culture, cells were lysed and protein profiled. Differentially expressed proteins by glucose or palmitate were identified by peptide mass fingerprinting. M_r _and pI denote relative molecular mass and isoelectric point of the protein.

**Table 3:** Glucose and palmitate-regulated INS-1E proteins

**Gene name**	**Primary accession no.**	**Ncbi accession no.**	**No. peptides matched**	**Seq cov (%)**	**Mowse score**
Calm1, Calm3	P62161	NP_114175.1 NP_036650.1	4	42	90
G6pdx, G6pd	P05370	NP_058702	29	40	176
Ganab		NP_001099804	19	25	191
Gars	Q5I0G4	AAH88347	19	40	155
Hnrnpa3, Hnrpa3	Q6URK4	NP_444493		23	105
Lonp1, Lon, Prss15	Q924S5	NP_596895	15	18	143
Ndufv1	Q5XIH3	NP_001006973	17	37	194
Pgk1, Pgk-1	P16617	NP_445743	15	52	131
Psmc5	O35050	BAA22935	17	41	190
Rab2	P05712	EDM11646	9	55	108
RGD1561681_predict.		XP_001054125	27	58	257
Tcp1, Cct1, Ccta	P28480	NP_036802	18	34	146

INS-1E cells were cultured at 5.5 or 20 mM glucose in the absence or presence of palmitate for 48 hours. After culture, cells were lysed and protein profiled. Differentially expressed proteins by glucose or palmitate were identified by peptide mass fingerprinting.

**Figure 3:**
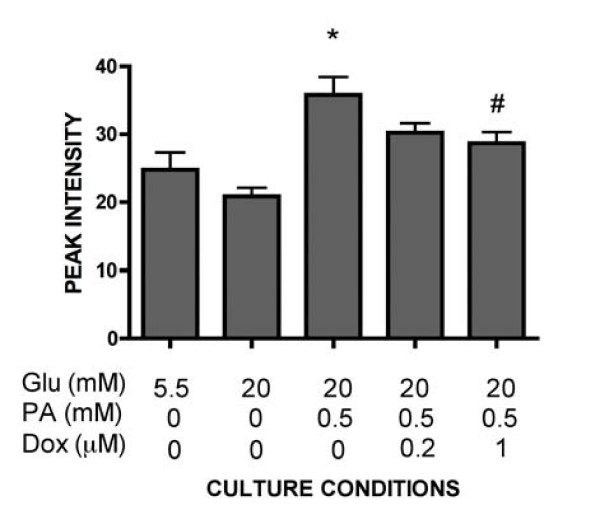
**Normalized expression of the 7.14 kDa protein in INS-1E cells by CPT1 over-expressing**. The 7.14 kDa protein was elevated in INS-1E cells cultured in the presence of 20 mM glucose and 0.5 mM palmitate. Enhanced CPT1-expression induced by 0.2 or 1 μM doxycycline, normalized the levels of the 7.14 kDa protein. Results are means ± SEM for six independent experiments. *P < 0.05 compared to cells cultured at 20 mM glucose, #p < 0.05 compared to non-induced INS-1E cells cultured in the presence of 20 mM glucose and palmitate.

We previously observed that basal insulin release was lowered in INS-1E cells cultured in the presence of 20 mM glucose and palmitate when CPT1 was over-expressed in a regulated manner [12]. To delineate mechanisms by which elevated CPT1-levels corrected basal insulin release, INS-1E cells cultured in the presence of 20 mM glucose and palmitate and expressing different levels of CPT1 were protein profiled using either protein array followed by SELDI-TOF-MS or 2D-PAGE. When searching for INS-1E proteins with expression levels correlating with CPT1 expression levels, a 7.14 kDa protein was found (Fig 3). This protein was induced in INS-1E cells cultured in the presence of 20 mM glucose and palmitate compared to cells cultured in the absence of palmitate. The 7.14 kDa protein was decreased to levels observed in control cells, when CPT1 was over-expressed. Since calmodulin was down-regulated in INS-1E cells exposed to palmitate (Fig 2A), we also measured amounts of calmodulin in INS-1E cells over-expressing CPT1. The peaks appearing at masses 16.8, 17.0 and 17.2 kDa were not altered in INS-1E with increased CPT1 levels, however (data not shown).

### Attenuation of stimulatory insulin release: differential INS-1E protein expression induced by high glucose

To elucidate mechanisms by which prolonged exposure to elevated glucose levels decrease stimulatory insulin release, INS-1E cells were cultured at 5.5 or 20 mM glucose. After culture, protein profiles were generated by SELDI-TOF-MS and 2D-PAGE. When stringent peak and spot detection criteria were applied, 34 differentially expressed proteins remained that were glucose-regulated. Whereas five proteins were discovered by the former approach, 29 proteins were detected by the latter approach (Table 4). Eleven proteins were identified as alpha glucosidase, gars, glucose-6-phosphate dehydrogenase, heterogenous nuclear ribonucleoprotein A3, lon peptidase, nicotineamide adenine dinucleotide hydrogen (NADH) dehydrogenase, phosphoglycerate kinase, proteasome p45, rab2, pyruvate kinase and t-complex protein (Table 2 and 3). All identified proteins were up-regulated by elevated glucose levels (Table 4) and their positions in the 2D-PAGE map were marked by their gene names (Fig 2B).

**Table 4:** Glucose-regulated INS-1E proteins

**Protein mass (kDa)**	**pI**	**Protein expression ratio G20/G5.5**
5.74		0.63

5.81		0.70

7.22		0.79

11.87		1.21

40.19		1.36

26 ^Rab2^	5.9	1.7

28	7.6	3.51

35	7.9	34.8

38	6.0	1.25

39	8.7	1.29

40 ^Hnrnpa3, Hnrpa3^	8.6	1.24

41	5.1	2.07

41	5.9	1.16

43 ^Pgk1, Pgk-1^	8.2	1.57

45 ^Psma6^	7.5	1.43

50	4.5	55.9

51 ^Ndufv1^	7.9	1.18

55	6.0	1.26

57	6.3	2.52

58 ^G6pdx, G6pd^	5.9	1.69

59 ^RGD1561681_predict.^	7.8	1.75

61 ^Tcp1, Cct1, Ccta^	5.8	1.53

61	5.8	1.79

63	5.0	1.51

63	5.8	1.16

65	6.0	1.31

69	5.9	1.27

71	6.0	1.80

72 ^Gars^	5.8	2.15

73	5.7	1.42

81	5.5	1.27

89 ^Ganab^	5.6	1.46

90 ^Ganab^	5.6	1.37

93 ^Lonp1, Lon, Prss15^	5.8	1.86

INS-1E cells were cultured at 5.5 or 20 mM glucose for 48 hours. After culture, cells were lysed and protein profiles generated by SELDI-TOF-MS using Q10-array or by 2D-PAGE. Masses of differentially expressed proteins obtained by SELDI-TOF-MS (5 first proteins) or by 2D-PAGE are listed. Gene names of identified proteins are indicated. Results are means ± SEM 4–6 independent experiments.

## Discussion

In the present study mechanism of aberrant GSIS were delineated by protein profiling of insulin-producing cells cultured at elevated levels of glucose and palmitate using SELDI-TOF-MS and 2D-PAGE.

INS-1E cells cultured at control condition of 5.5 mM glucose responded to stimulatory glucose concentration, GSIS, with approximately 10-fold enhanced insulin secretion over a 30-min period compared to basal insulin secretion, which is comparable to the secretory response of human islets [16,17]. However, in individuals with T2DM, GSIS is severely reduced or even absent [2,17,18]. Such alterations in the dynamic range of insulin levels are the result of lowered insulin release at stimulatory glucose concentrations combined or not with elevated insulin secretion at non-stimulatory glucose levels, the basal insulin secretion. Whereas absent initial rise of insulin release is a hallmark of T2DM [2,18,19], elevated basal insulin release is characteristic of T2DM individuals with insulin resistance [20]. The latter individuals display both hyperglycemia and hyperlipidemia, which made us investigate how elevated levels of glucose and fatty acid palmitate affected the insulin-producing cell. Palmitate was chosen based on its effects of elevating basal insulin release [3,4]. In addition, the fatty acid is associated with particularly β-cell detrimental effects, where the underlying causes have only partly been defined [4,6-10].

When episodes of hyperglycemia are not extended, granular exocytosis is balanced by formation of new insulin granules resulting in maintained insulin content [21]. In the present study, insulin content was severely reduced after extended hyperglycemia similar to what was previously observed in INS-1E cells and primary islets [22,23]. In contrast, palmitate did not affect insulin content. De-granulation has been suggested to be a main contributor to the loss of GSIS. However, cells cultured in the presence of 20 mM glucose and palmitate released similar amounts of insulin in response to a stimulatory glucose concentration as control cells, despite reduced insulin content. We concluded that other mechanism than lowered amounts of insulin granules must also be operational in the aberrant insulin release observed from β-cells cultured at elevated glucose alone or in combination with palmitate. To address mechanisms responsible for the inability to maintain hormonal stores and to delineate other processes contributing to the aberrant insulin release, glucose- and palmitate-regulated β-cell proteins were identified by protein profiling.

Profiling was conducted with SELDI-TOF-MS and 2D-PAGE to address how elevated levels of glucose and palmitate affected the β-cell proteome. The combination of the approaches was triggered by the preferential detection of proteins in the low molecular range by SELDI-TOF-MS and high molecular range by 2D-PAGE [14,24]. Indeed, in the present study more than 80% of the peaks/spots detected by SELDI-TOF-MS or 2D-PAGE were in the 4–20 kDa or 20–150 kDa range, respectively. Among these peaks and spots differential expression was discovered.

To elucidate mechanisms by which elevated levels of the nutrients incur alterations in β-cell secretory function, the identification of the differentially expressed proteins is essential. Whereas such proteins are inherently separated by 2D-PAGE and can be subjected to PMF, differentially expressed proteins obtained by SELDI-TOF-MS need first to be separated and enriched. The higher number of identified proteins derived from the 2D-PAGE approach reflects this condition. On the other hand when changes in complex protein patterns are studied and the identities of the components are not demanded, SELDI-TOF-MS is superior to 2D-PAGE [15]. In addition, the amount of cells required to generate mass spectra and gels by the two approaches, respectively, was about two orders of magnitude higher when utilizing 2D-PAGE. Although not a consideration when profiling cell lines, the enhanced sensitivity in SELDI-TOF-MS is crucial when analyzing protein patterns of scarce tissue e.g. human islets [15].

In the present study, several proteins were differentially expressed in response to elevated levels of glucose. The up-regulation of glycolytic enzymes phosphoglycerate kinase 1 and pyruvate kinase 3 together with enhanced expression of NADH dehydrogenase flavoprotein 1 of complex I of the respiratory chain indicate enhanced glucose metabolism. Under such conditions formation of reactive oxygen species (ROS) is enhanced [25]. It was therefore not surprising that glucose-6-phosphate dehydrogenase was up-regulated. This enzyme is induced by elevated ROS and determines production of nicotineamide adenine dinucleotide phosphate hydrogen (NADPH) via shuttling of glucose through the pentose phosphate shunt. All major ROS-metabolic enzymes, directly or indirectly depend on NADPH [26]. The glucose-induced proteins glycyl-tRNA synthetase (gars) and heterogenous nuclear ribonucleoprotein A3 (hnrnp A3) both serve regulatory functions in protein translation. Whereas gars catalyzes the synthesis of glycyl-tRNA, which is required to insert glycine into proteins [27], hnrnp A3 is involved in pre-mRNA processing, transcriptional regulation, recombination and telomere maintenance [28]. Further effects on protein synthesis were indicated by the up-regulated endoplasmic reticulum (ER)-associated alpha glucosidase 2 and cytoplasmic t-complex protein 1. Both proteins take part in protein folding where the former enzyme is an important part of the calnexin/calreticulin protein synthesis quality control system [29] and the latter acts as a chaperone with folding capacity [30]. The importance of glucose-induced ROS and its negative effects on proteins was also indicated by up-regulation of the lon protein, which is a mitochondrial ATP-dependent protease engaged in mitochondrial protein degradation by recognizing unfolded proteins [31,32].

However, proteasomal degradation may also be enhanced as indicated by the rise in proteasome p45. In this context accumulation of insulin and other granular proteins in the ER may play a role as indicated by the glucose-induced up-regulation of rab2. This small GTPase decreases vesicular transport between the ER and the Golgi complex when induced [33]. Indeed, glucose-induced rab2 with ensuing impaired anterograde transport of secretory granule protein precursors may contribute to explain the observed decrease in GSIS and insulin content. The latter findings are in contrast to when islets were exposed to elevated glucose levels for a shorter time period [14]. When such islets were protein profiled manifestations of enhanced granular formation maturation and trafficking were obtained. The lowered hormonal levels obtained in cells exposed to prolonged elevated glucose levels indicate that protein degradation is surpassing synthesis, where the latter may even be attenuated. Such cellular reactions are observed as manifestations of the unfolded protein response (UPR), which is a cellular adaptive response systems initiated when the protein synthesis machinery is compromised [34]. Indeed, initiation of the UPR has been observed in β-cells exposed to prolonged elevated glucose levels [35]. In such cells, induction of lipogenic enzymes resulting in lipid accumulation was proposed to explain the impaired GSIS. When the lipogenic expression was decreased, GSIS was improved. The improvement of the stimulatory secretory levels in the present study when including the fatty acid palmitate during the acute exposure may therefore be explained by maintaining fatty acid cellular levels, which are critical for optimal GSIS [36].

Increased basal insulin release was observed when cells were cultured in the presence of palmitate, confirming previous studies in INS-1E cells [4,12,37] and isolated islets [3]. Several β-cell proteins were regulated by palmitate. One of these proteins was identified as calmodulin. This protein is likely to undergo post-translational modifications such as phosphorylation [38], which may account for the three separate peaks observed. Calmodulin in INS-1E cells was lowered when palmitate was added to culture medium containing 20 mM glucose. Its role in insulin secretion is pleiotropic including regulation of the cytoplasmic Ca^2+ ^concentration by controlling Ca^2+^-ATPases of the plasma membrane and the ER [39,40], which has effects on mobilization and exocytosis of insulin granules [41,42]. In addition, calmodulin is regulating kinase and phospahatase activities via Ca^2+^/calmodulin-dependent protein kinases and calcineurin, respectively [43,44]. In a previous study, we observed that palmitate-induced rise in basal insulin secretion was counteracted in INS-1E cells over-expressing CPT1 in a regulated manner [12]. The role of calmodulin for this partial normalization of basal insulin release appears to be minimal since calmodulin levels were not affected by over-expressing the fatty acid transporter. Instead, over-expression of CPT1 normalized a 7.14 kDa protein, which was highly elevated in cells exposed to augmented levels of palmitate or glucose. The beneficial effects of CPT1 over-expression was accompanied by alleviation of apoptosis and attenuation of UPR-markers phosphorylated eIF2α and pro-apoptotic CHOP/GADD153 [12]. Factors contributing to elevated basal insulin secretion and palmitate-induced alterations in GSIS have instead been attributed to altered expression of enzymes of glucose metabolism including glucokinase, phosphofructokinase and pyruvate dehydrogenase, and of the fatty acid receptor GPR40 [45-47].

## Conclusion

In conclusion, differentially expressed proteins were identified by protein profiling of insulin-producing β-cells exposed to prolonged elevated glucose and palmitate levels. The identified proteins may represent mechanisms involved in islet functional deterioration in T2DM.

## Methods

### Chemicals and reagents

Reagents of analytical grade and MilliQ water were used. Glucose, HEPES, insulin peroxidase, DTT, CHAPS, TrizmaBase, sinapinic acid, sodium pyruvate, glutamine, penicillin, streptomycin, sodium salt of palmitate and doxycycline were obtained from Sigma (St. Lous, MO). BSA fraction V (fatty acid free) was purchased from Boehringer Mannheim GmbH (Mannheim, Germany). Urea, 2-D Quant Kit and Pharmalyte 3 to 10 were purchased from GE Healthcare (Uppsala, Sweden) and ASB14 from Calbiochem (San Diego, CA). Alpha-cyano-4-hydroxy-cinnamic acid (CHCA), immobilised pH gradient (IPG) Ready Strips (11 cm), peptide/protein molecular weight standards, pH range 3–10 non-linear, ReadyPrep 2-D Cleanup Kit, 2-D SDS-PAGE Standards and 12.5% precast polyacrylamide gels (Criterion Gel System) were purchased from Bio-Rad (Hercules, CA). PEFA-bloc^®^, acetonitrile, formic acid, trifluoroacetic acid and octyl-β-D-Glucopyranoside, thiourea, Triton X-100, ammonium hydroxide, citric acid monohydrate, glacial acetic acid, methanol and Tris were purchased from Merck (Darmstadt, Germany). Trypsin was obtained from Roche Diagnostics (Indianapolis, IN). RPMI 1640 culture medium, fetal bovine serum (FBS), sodium pyruvate, glutamine, penicillin, streptomycin were purchased from Invitrogen (Carlsbad, CA). Culture flasks and culture plates were obtained from BD Biosciences Labware (Franklin Lakes, NJ). PageBlue protein staining solution and prestained protein ladder were purchased from Fermentas (Vilnius, Lithuania). The rat insulin standard was obtained from Novo Nordisk (Bagsvaerd, Denmark). Guinea pig anti-mouse insulin antibodies were produced in our laboratory. IgG certified 96-well microtiter plates were purchased from Nunc (Roskilde, Denmark).

### Cell culture

Rat insulinoma INS-1E cells, kindly donated by Dr. Pierre Maechler, Geneva University [48], were cultured between passages 76–90 in a humidified atmosphere containing 5% CO_2 _in RPMI 1640 medium supplemented with 10 mM HEPES, 10% (v/v) heat-inactivated FCS, 2 mM glutamine, 100 U/ml penicillin, 100 μg/ml streptomycin, 1 mM sodium pyruvate and 50 μM β-mercaptoethanol. Cells were exposed to 5.5 or 20 mM glucose in the absence of presence of 0.5 mM palmitate for 48 hours. Fatty acid stock solution of palmitate (100 mM) was prepared in 50% ethanol. This stock solution was diluted in the culture medium to a final concentration of 0.5 mM and then allowed to complex with 0.5% fatty acid-free BSA. The CPT-1 over-expression was performed with a regulated adenoviral construct as previously described [12].

### Measurements of insulin secretion and insulin content

After culture, GSIS was measured from INS-1E cells as previously described [12]. The cells were maintained for 1 hour at 37°C in culture medium containing 3 mM glucose in the presence or absence of palmitate followed by another 1 hour incubation in buffer containing 0.5% fatty acid free BSA and (in mM): NaCl 125, KCl 5.9, MgCl_2 _1.2, CaCl_2 _1.28, HEPES 25, glucose 3 and palmitate 0 or 0.5 titrated to pH 7.4 with NaOH. GSIS was measured from INS-1E cells incubated for 30 min at 37°C in similarly composed buffer with either 3 or 20 mM glucose in the absence or presence of palmitate, respectively. After incubation, supernatants were collected and stored at -20°C until assayed by enzyme-linked immuno-sorbent assay (ELISA) [49]. INS-1E cells were subsequently washed in a similar buffer as used for insulin release experiments without BSA, lysed in 0.1% Triton X-100 and 25 mM NaOH and stored at -20°C until assayed for total protein or insulin content measurements by DC protein assay (BioRad) and ELISA [49], respectively. Insulin release and content were normalized to total protein.

### Sample preparation and protein profiling

#### SELDI

INS-1E cells samples were prepared and protein profiled essentially as described previously [13]. The cells lysed in buffer consisting of 50 mM Trizmabase, 8 M Urea, 3% CHAPS, 1% ASB14 and 5 mM PEFA-bloc and diluted three times with 50 mM Trizmabase. Total protein content was determined with Lowry assay (BioRad, Hercules, CA) and the samples were stored at -80°C until analysis. Protein arrays with a chromatographic surface of anionic exchanger, Q10 (Bio-Rad), were used for profiling. Samples were diluted in binding buffer consisting of 0.1 M phosphate buffer, pH 7.0, supplemented with 0.1% Triton X-100 to a protein concentration of 0.1 μg/μl and applied on the protein arrays. Arrays with sample and peptide/protein molecular weight standards were read in a PBSII (Bio-Rad). Generated mass spectrograms were analyzed using the supplied software (Bio-Rad).

#### Two-dimensional gel electrophoresis

Samples were prepared and protein profiled essentially as described previously [14]. INS-1E cells were lysed in buffer containing 1% Triton X-100, 1% SDS and protease inhibitor cocktail, homogenized by sonication, which was followed by treatment with ReadyPrep 2-D cleanup Kit. The protein pellet was re-suspended in rehydration solution containing 7 M urea, 2 M thiourea, 0.5% Triton X-100, 4% CHAPS, 0.5% pharmalyte (pH 3–10), 0.1% NP-7 and 60 mM DTT for the iso-electric focusing (IEF) and protein concentration was determined using a 2-D Quant Kit. The protein amount per gel was 250 μg.

Individual 11-cm IPG strips, pH 3–10 NL, were rehydrated in 185 μl of sample at 20°C for 15 hours. The rehydrated strips were then focused on the Protean IEF Cell (Bio-Rad) for about 35 kV·h at a maximum of 8.0 kV in rapid voltage ramping mode with a maximum current per strip of 50 μA. The equilibration of the IPG strips after IEF was performed by reduction in a solution containing 1% DTT, 6 M urea, 2% SDS, 30% glycerol, 0.002% Coomassie Brilliant Blue R-250 (CBB) and 0.05 M Tris pH 8.8 followed by alkylation in a solution containing 6 M Urea, 2% SDS, 30% glycerol, 0.002% CBB, 0.05 M Tris pH 8.8 and 4% iodoacetamide.

Protein mass separation was performed by electrophoresis using 12.5% precast SDS-polyacrylamide gels, which were stained with Pageblue protein staining solution overnight and imaged using a GS-800-calibrated densitometer (Bio-Rad). Raw scans were processed quantitatively, qualitatively and statistically analyzed by the 2-D gel analysis software, PDQuest (Bio-Rad). For between-gel comparisons, a set of spot generation conditions was used. The quantity of each spot was normalized by the total intensity of valid spots to minimize the effect of experimental factors on protein spots. All the quantitatively different spots were also verified manually.

### Protein purification and identification

The identities of differentially expressed proteins discovered by SELDI-TOF-MS or 2D-PAGE were obtained by peptide mass fingerprinting (PMF) essentially as described previously [14,18]. Proteins discovered by SELD-TOF MS had to be purified by anion exchange (Vivapure Q Mini spin columns, Vivascience, Hannover, Germany) and hydrophobic fractionation (Polymer Laboratories, Darmstadt, Germany), separated by 18% SDS-PAGE and stained in colloidal Coomassie stainer over night prior to PMF. Purified and separated proteins were verified to correspond with the SELDI-TOF-MS discovered proteins by applying passively eluted proteins from the gel pieces on NP20-chip. Gel pieces containing relevant proteins, discovered by either SELDI-TOF-MS or 2D-PAGE, were in-gel digested followed by mass determination of the tryptic fragments recorded on an Applied Biosystems Q-STAR XL with a ProteinChip Interface (PCI-1000) Q-star MALDI-TOF mass spectrometer for proteins discovered by SEDLI-TOF MS or a Bruker Reflex IV (Bruker Daltonics, Bremen, Germany) MALDI-TOF mass spectrometer for proteins discovered by 2D-PAGE. All mass spectra were internally calibrated with trypsin autolysis products and masses of known contaminants e.g. keratin were removed. Proteins were identified by PMF with the search program Mascot (Matrix Science, London, UK). The criteria used to accept identifications included the molecular weight search (MOWSE) score (protein scores greater than 61 was considered significant) and whether the theoretical molecular weight ± 20% and p*I *of the matched protein were within the experimental p*I *value ± 1.00. SWISS-PROT was used as the protein sequence database and the peptide masses were compared to the theoretical peptide masses of all available proteins from *Rattus norvegicus*. Information about the identified protein and its putative function was found at the ExPASy Molecular Biology Server at SWISS-PROT  and at the National Centre for Biotechnology Information (NCBI), which were accessed between June–July 2008.

### Statistical analysis

The insulin release data are represented as means ± SEM for four to six independent experiments. Differences between groups are assessed by one-way ANOVA followed by Tukey's post hoc test. Probability level of p < 0.05 was considered to be statistically significant. Significant changes between the protein profiles for four to seven independent experiments were determined using Student's *t*-test.

## Competing interests

The authors declare that they have no competing interests.

## Authors' contributions

EMS was responsible for design, planning, carrying out cell culture, sample preparations, obtaining protein profiles with SELDI-TOF-MS and 2D-PAGE. EMS also performed the data/statistical analysis, protein purification and identification of proteins obtained from SELDI-TOF-MS and contributed to write the manuscript. MH carried out the data/statistical analysis for the spots from 2D-PAGE. PB conceived the study, participated in its design and was responsible for writing the manuscript. All authors read and approved the final manuscript.
